# Level of Awareness of Mammography among Breast Cancer Patients Attending Follow-up at a Tertiary Hospital, Addis Ababa, Ethiopia

**DOI:** 10.4314/ejhs.v32i1.9S

**Published:** 2022-10

**Authors:** Abdi Alemayehu Duguma, Teklehaimanot Mezgebe Nguse, Seife Teferi Dellie, Daniel Admassie Tadesse

**Affiliations:** Department of Radiology, College of Health Sciences, Addis Ababa University

**Keywords:** Breast, cancer, breast cancer, awareness, mammography

## Abstract

**Background:**

Breast cancer is the most common cancer type and the most common cancer related cause of death worldwide in women. This study was aimed to assess the awareness of mammography among breast cancer patients attending follow-up at Tikur Anbessa Specialized Hospital, Addis Ababa, Ethiopia.

**Methods:**

Hospital based cross sectional study was conducted from July 01 to August 30, 2017 at Tikur Anbessa Specialized Hospital. Interviewer administered questionnaire was used to obtain data from 270 breast cancer patients who came to radiology department for follow up imaging. Collected data were checked for completeness, coded and entered into Epi Info 3.1 software and exported to SPSS Version 21 for analysis.

**Results:**

Two hundred and twenty four (83%) of the study participants had no awareness of mammography while 46 (17%) of them had awareness of mammography. Out of the total 270 women, only 38(14.1%) had mammography test. In multivariate logistic regression analysis; level of education was found to have significant association with awareness of mammography. Women with breast cancer who completed secondary education were 4.5 times (AOR= 4.5; 95% CI: 1.39, 14.77) more likely to have awareness of mammography compared to women with breast cancer who were illiterate.

**Conclusion:**

This study revealed low awareness, knowledge and practice of mammography among women with breast cancer. Knowledge of participants about risk factor for breast cancer was also low.

## Introduction

Cancer is a group of diseases characterized by the uncontrolled growth and spread of abnormal cells. If the spread is not controlled, it can result in death (1). Globally, breast cancer is the most common cancer among women, comprising 23% of the female cancers. It is also the primary cause of cancer-related deaths in low-income countries (1,2). One in eight women born today will be diagnosed with breast cancer at some time in her life (2).

In 2012 there was 1.7 million new case of breast cancer diagnosed. This represents 12% of all new cases of cancer and 25% of new cancer cases in the women (3). In sub-Saharan Africa, breast cancer is responsible for one in four diagnosed cancers and one in five cancer deaths in women(4). Despite its emerging public health importance, incidence rates are still generally low in Africa, below 35 per 100,000 women in most countries as compared to over 90–120 per 100,000 women in most European countries.

Study conducted in Spain showed that the awareness of mammography in the general population is 95.03 % (5). Another study conducted on women' attending health facility in Brazil showed that the awareness of mammography was 93.5% and their health centers were the main instrument to provide this knowledge (6). In a study done in Delhi city, India only four participants had not heard about the term mammography before (7). According to study done in Abidan city of Nigeria, only 5% of women’ attending health care facility have ever heard about mammography. One study conducted in Uganda indicates 71% women attending health facility had never heard about screening mammography (8).

Breast cancer is the second most often-occurring cancer next to cervical cancer among women in Ethiopia (9). A retrospective study conducted in Tikur Anbessa Specialized Hospital from 1997–2012 indicates that from total of 16,622 new cases registered, 3460 were new cases of breast cancer with prevalence of 20.8% (3). Study conducted on female health professionals working in the government Hospitals of Addis Ababa, awareness of mammography as screening method for breast cancer was 81.4 % and 84% of study participants never had mammography screening test (10).

Many of the studies previously done in Ethiopia focused on knowledge, attitude and practice of breast cancer risk factors and screening methods. Some of the studies conducted before mainly focused on female health professionals, nurses, female students and teachers. Therefore, this study was aimed to assess the awareness of mammography among breast cancer patients attending follow-up at Tikur Anbessa Specialized Hospital, Addis Ababa, Ethiopia.

## Materials and Methods

**Study Setting**: The study was conducted at Radiology Department, Tikur Anbessa Specialized Hospital (TASH), Addis Ababa, Ethiopia. TASH is the largest referral teaching hospital, under the administration of Addis Ababa University. The hospital has 700 beds and gives diagnostic and treatment service for about 370,000–400,000 patients per year. Radiology department is one of the many departments in the institution which gives radiologic medical service and academic activities.

**Study design**: Hospital-based descriptive cross-sectional study using interviewer-administered questionnaire was conducted from July 01 to August 30, 2017.

**Participants**: The source population was all breast cancer patients who had follow-up at oncology department in TASH. All breast cancer patients who came to radiology department for follow-up imaging during the study period were the study population. The inclusion criterion was breast cancer patients who had treatment and follow up at TASH. Patients who were not enrolled at TASH; severely ill and non-communicative patients were excluded from study.

**Study variables**: The dependent variables in this study were awareness of mammography and practice of mammography screening. The independent variables in this study were sociodemographic characteristics such as age, marital status, level of education and employment.

**Sample size determination**: The sample size was calculated to estimate the proportion of breast cancer patients having awareness of mammography with 95% confidence level and 5% error bound. The prevalence of breast cancer in TASH was about 20.8 % (3). Accordingly, the calculated sample size was 253 (n= 253). To compensate non-response and missing data, there was a need of oversample it by 10% of the computed sample size. Therefore, the final number of breast cancer patients who were included in this study was 278.

**Data collection and management**: Interviewer-administered questionnaire was used to obtain information from the patients about their sociodemographic characteristics as well as their awareness, knowledge and utilization of breast cancer screening services. Data was collected by radiology residents after adequate information about the study and contents of questionnaire were given. To ensure quality of data, pre-test of data collection tool was done on patients not included in the main study by taking 5% respondents of the total sample size.

**Data processing and analysis**: Collected data were checked for completeness, coded and entered into Epi Info 3.1 software and exported to SPSS Version 21 statistical software for analysis. Binary logistic regression was used to explore possible associations between the dependent variable and independent variables.

**The following operational definition are used**

**Have awareness**: Those patients who had heard of mammography

**Have no awareness**: Those patients who had not heard of mammography

**Knowledge**: To categorize the knowledge of women regarding risk factors of breast cancer, from the given six risk factors, those mentioned > 4 were considered as having good knowledge, 3–4 as fair knowledge and those mentioned ≤ 2 were considered to have poor knowledge of risk factor. As to knowledge score of mammography; those who scored above mean were considered to have good knowledge and those who scored below the mean were considered to have poor knowledge

**Ethical consideration**: Data collection was started after getting permission from the ethical review committee of TASH. The purposes and the importance of the study was clearly explained by the data collectors when the study participant came for imaging and informed consent was obtained from each participant. To maintain confidentiality and anonymity, data collector was recruited from radiology department resident students.

## Results

**Socio-demographic characteristics of the respondents**: From a total of 278 breast cancer patients, 270 questionnaires were ready for analysis which made the response rate 97%. The mean age of breast cancer patients who participated in this study was 44.3 years and a range of 18–85 years. Majority of the participants in the study were aged between 40 to 49 years which accounted 72 (26.7%) followed by age range of 30–39 which was 61 (22.6%). Majority of the participants, 75 (27.8%) did not have formal education. Those with primary education, secondary education and tertiary education were 59 (21.9%), 74 (27.4) and 62 (23%), respectively. Larger proportions, 203(75.2%) were not employed. The majority of participants (84.4%) were married and 11.5% were single. Only 33 (12.2%) of the women had family history of breast cancer (Table 1).

**Table 1:** Socio-demographic characteristics of women with breast cancer in Tikur Anbessa Specialized Hospital, Addis Ababa, Ethiopia, 2017 (n = 270)

Variables	Frequency	Percent
**Age**		
<30	43	15.9
30–39	61	22.6
40–49	72	26.7
50–59	51	18.9
≥ 60	43	15.9
**Educational level**		
None	75	27.8
Primary education	59	21.9
Secondary education	74	27.4
Tertiary education	62	23
**Employment status**		
Employed	67	24.8
Not employed	203	75.2
**Marital status**		
Single	31	11.5
Married	228	84.4
Divorced	7	2.6
Widowed	4	1.5
**Family history of breast cancer**		
Yes	33	12.2
No	237	87.8

**Awareness of study participants about mammography**: Most of the respondents, 224 (83%) had no awareness of mammography while 46 (17%) of them had awareness of mammography. Thirty four (73.9%) heard of mammography after they diagnosed to have breast cancer and only 12 (26.1 %) women heard before they diagnosed to have breast cancer. Health facility was the major source of information 33 (71.7%) while the remaining 13 (28.3 %) mentioned television, radio, friends and neighbors as source of information about mammography. Among breast cancer patients who have heard of mammography, 23(50%) correctly answered that mammography as x-ray of breast. Half (50%) of them who have heard of mammography also knew the purpose of mammography. Majority of them, 42(91.3%) did not know age at which base line screening mammography should be started. Only three (6.5%) knew that screening mammography is done every year (Table 2). Among women who have mammography awareness only 10.4% have good knowledge about mammography and large proportion of them have poor knowledge.

**Table 2:** Awareness of women about mammography in Tikur Anbessa Hospital, Addis Ababa, Ethiopia, 2017 (n = 270)

Variables	Frequency	Percent
**Have you heard of mammography**		
Yes	46	17
No	224	83
**Source of information**		
Radio/ television	11	23.9
Health facility	33	71.7
Neighbors or friend	2	4.4
**When you heard about mammography**		
Before diagnosed to have breast cancer	12	26.1
After or at the time when diagnosed to have breast cancer	34	73.9
**What is mammography**		
X-ray of breast	23	50
Do not know	23	50
**Purpose of mammography**		
To diagnose breast cancer	23	50
I do not know	23	50
**Age at which first mammogram should be done**		
Whenever problem in the breast	2	4.3
Above 40 year	2	4.3
I do not know	42	91.3
**Recommended frequency for mammography screening test**		
Once a year	3	6.5
I do not know	43	93.5
**Is mammogram same as ultrasound**		
Yes	3	6.5
No	9	19.5
I do not know	34	73.9

**Practice of mammography**: Out of the total 270 women, only 38(14.1%) had mammography test. Among this 37(97.3%) were diagnostic mammography and only one (2.7%) patient had screening mammography test. Majority of them, 47.8% women whose age were above 40 years reported that they do not know that screening mammography test was supposed to be done. Other reasons mentioned for not having screening mammography were lack of advice from physician, non-availability of the procedure and lack of knowledge about screening mammography (Table 3).

**Table 3:** Practice of mammography among women with breast cancer in Tikur Anbessa Hospital, Addis Ababa, Ethiopia, 2017 (n = 270)

Variable	Frequency	Percent
**Have you had mammography test**		
Yes	38	14.1
No	232	85.9
**Purpose of the test**		
Diagnostic mammography	37	97.3
Screening mammography test	1	2.7
**Reason for not having mammography**		
Don't know that it was supposed to be done	129	53.4
No physician told me to do so	56	23.2
It is not available to get the procedure	41	16.9
Not required as there was no problem of breast	12	4.9
I didn't know where to go	4	1.6

**Factors associated with awareness of mammography**: In bivariate logistic regression analysis, level of education (secondary and tertiary), marital status (divorced) and good knowledge of risk factor for breast cancer had association with awareness of mammography. Women with breast cancer those having tertiary education were 5.7 times (COR=5.7; 95% CI: 1.9, 16.53) more likely to have awareness of mammography compared to women with breast cancer who had no formal education. Regarding marital status, divorced women were 7 times (COR=7; 95% CI: 1.03, 47.4) more likely to have awareness of mammography compared to women with breast cancer those single. Women with breast cancer who had good knowledge for risk factor of breast cancer were 6.7 times (COR=6.7; 95% CI: 1.7, 26), more likely to have awareness of mammography compared to women with breast cancer who had poor knowledge of the risk factors for breast cancer. In multivariate logistic regression analysis; only level of education (secondary and tertiary), was found to have statistically significant association with awareness of mammography. Women with breast cancer those who completed secondary education were 4.5 times (AOR=4.5; 95% CI: 1.39, 14.77) more likely to have awareness of mammography compared to women with breast cancer who were illiterate (Table 4).

**Table 4:** Bivariate and multivariate logistic regression analysis of mammography awareness and its explanatory variables Tikur Anbessa Hospital, Addis Ababa, Ethiopia, 2017 (n = 270)

Variables	Awareness		P	COR (95% CI)	AOR (95% CI)
	Yes	No	Value		
Age					
<30 years	1(2.2%)	19(8.5%)		1.0	1.0
30–39 years	15(32.6%)	53(23.7%)	0.23	0.2 (0.03, 2.20)	3.7.(0.41,33.58)
40–49 years	14(30.4%)	64(28.6%)	0.43	1.3 (0.53, 3.57)	2.43(0.25, 22.91)
50–59 years	8(17.4%)	49(21.9%)	0.34	1.0 (0.41, 2.77)	2.98(0.30, 28.89)
≥60 years	8(17.4%)	39(17.4%)	0.22	0.79(0.27,2.31)	4.24(0.41, 43.99)
Level of education					
None	5(10.9%)	70(31.3%)		1.0	1.0
Primary	9(19.6%)	50(22.3%)	0.10	2.5 (0.79, 7.9)	2.77(0.82, 9.33)
Secondary	14(30.4%)	60(26.8%)	0.01	3.2 (1.1, 9.5)*	4.54(1.39,14.77)*
Tertiary	18(39.1%)	44(19.6%)	0.001	5.7 (1.9,16.53)*	8.16(2.24, 29.70)*
Employment status					
Employed	12(26.1%)	55(24.6%)	0.13	1.08 (0.52, 2.23)	0.50(0.20, 1.22)
Not employed	34(73.9%)	169(75.4%)		1.0	1.0
Marital status					
Single	3(6.5%)	28(12.5%)		1.0	1.0
Married	39(84.8%)	189(84.4%)	0.57	1.9(0.55,6.65)	1.58(0.32, 7.87)
Divorced	3(6.5%)	4(1.8%)	0.14	**7 (1.03, 47.4)** *	5.66(0.54,59.15)
Widowed	1(2.2%)	3(1.3%)	0.36	3.1(0.24,40.1)	3.86(0.20,73.31)
Number of pregnancy					
None	6(13.0%)	44(19.6%)		1.0	1.0
One	19(41.3%)	57(25.4%)	0.91	1.44 (0.9, 6.63)	1.07(0.29, 3.93)
Two	8(17.8%)	46(20.5%)	0.71	1.2 (0.40, 3.97)	0.77(0.19,3.07)
Three and above	13(28.3%)	77(34.4%)	1.0	1.2 (0.43, 3.48)	1.0(0.27, 3.70)
Family history of breast	cancer				
Yes	4(8.7%)	29(12.9%)		1.0	1.0
No	42(91.3%)	195(87.1%)	0.54	1.56 (0.52, 4.67)	1.43(0.42, 5.03)
Good knowledge of risk factor					
Yes	5(10.9%)	4(1.8%)	0.09	**6.7 (1.7, 26.0)** *	3.72(0.79, 17.44)
No	41(89.1%)	220(98.2%)		1.0	1.0

* **P**: value is significant at P < 0.05, **COR**: Crude Odd Ratio, **AOR**: Adjusted Odd Ratio

## Discussion

This study revealed, 224 (83%) had no awareness of mammography, while 46 (17%) of them had awareness of mammography. In contrast to this study, a study conducted in Addis Ababa among female health workers indicated that 342 (81.4%) had awareness of mammography as a screening method for breast cancer (10). The difference can be explained by difference in sample size, composition of study population, educational level, access to health care and information like mass Medias.

This study revealed that the proportion of patients who had ever heard of mammography was 46 (17%). This figure is higher than proportion reported among women of Nigeria found that only 5% of women is attending outpatient clinic had mammography awareness (11). This difference is a reflection of the high level of education of the samples studied in this study where 136 (50.30%) of the samples had secondary and tertiary level of education compared to 36 (14%) in the study conducted in Nigeria. About 74% of patients having mammography awareness in current study ever heard of mammography after or at the time of their breast cancer diagnosis. However, in this study awareness level was inconsistent with that of developed and middle-income countries. Study conducted in India (7) indicates high proportion of mammography awareness, which accounts 99%. This difference can be explained by difference in level of education of study participant, where 55.5% of participants in the Indian study were women is of University graduate while in the current study 25% of the patients have tertiary level education.

This study showed that only 10.4% of the study participants had good knowledge of mammography among patients who had mammography awareness. In this study, 50% of patients who had mammography awareness knew that mammography is x-ray of breast with the purpose of diagnosing breast cancer. Study from India (12) showed that about 56% of the participant knew what mammography is and 27.3% knew the purpose of mammography. This difference in knowledge of purpose of mammography can be explained by large proportion of the patients in the current study get awareness of mammography after diagnosed to have mammography. In this study, only 4.3% of patients who had mammography awareness know screening mammography start at the age of 40 and only 6.3% know it is done every year. This is lower than the study conducted in India (12) where 11.8% and 21.6 of participant women' knew age at which base line mammography should be done and frequency to done respectively. This difference can be explained by difference in composition of study population and difference in educational level.

In the current study practice of mammography test was about 14 % and 97.3% of the test was for diagnostic purpose while only 2.7% was for screening purpose. This result is better than studies done in Nigerian (11) and Uganda (8) where none of the participant had mammography test before. This difference could be due to variability in composition of study population. Furthermore, this finding was inconsistent with the finding in Brazil where 38% of women were practiced mammography screening test (6). The explanation for this difference may be due to the high level of knowledge about mammography in the Brazil study with proportion of 93% compared with low level of knowledge in the current study, nearly 10%.

The findings from association between mammography awareness and independent variables revealed that education was significantly associated with mammography awareness. Having tertiary level of education (AOR=8.1; 95% CI: 2.24, 29.7) was significantly associated with awareness of mammography. Obajimi et al. (12) also showed similar association of awareness of mammography and level of education. Similar finding was also reported by Akinola (13) and Lee et al. (14). Educated women are more likely to benefit from most messages concerning breast cancer knowledge and methods of prevention and thus more likely to learn about mammography. The same finding was reported in the study done in United Arab Emirates among women where maternal education was significantly associated with the awareness of mammography (15).

Although this study has a significant input for researchers and policy makers, there is a limitation in that it only considered those who came to the health facility. This study revealed low awareness of mammography among women with breast cancer, which accounted about 17%. This study also indicated that only 14.1 % participants had practice of mammography and almost all was for diagnostic purpose with only one patient underwent screening mammography before. Level of education was significantly associated with awareness of mammography among women with breast cancer. Thus, there is a need to design awareness creation program by using different means like mass media and campaign to increase awareness and practice of women regarding mammography-screening test. Breast cancer patients come from different corner of the country; as TASH is the only oncology center in the country. Therefore, health care providers can play important role in dissemination of information related to breast cancer and early detection mechanism like screening mammography.
